# Finite element analysis of the lens profile during accommodation

**DOI:** 10.1371/journal.pone.0317740

**Published:** 2025-03-17

**Authors:** Ronald A. Schachar, Ira H. Schachar, Xiaomeng Li, Yutian Pu, Shubham Kumar, Farhad Kamangar, Boyd Hunter, Barbara K. Pierscionek, Pamela C. Cosman, Kehao Wang

**Affiliations:** 1 Department of Physics, University of Texas at Arlington, Arlington, Texas, United States of America,; 2 North Bay Vitreoretinal Consultants, Santa Rosa, California, United States of America,; 3 Beijing Advanced Innovation Center for Biomedical Engineering, School of Engineering Medicine, Beihang University, Beijing, China,; 4 Department of Electrical and Computer Engineering, University of California San Diego, San Diego, California, United States of America,; 5 Department of Computer Science and Engineering, University of Texas at Arlington, Arlington, Texas, United States of America,; 6 Praxis Optics, Elmira, New York, United States of America,; 7 Faculty of Health, Medicine and Social Care, Medical Technology Research Centre Anglia Ruskin University, Chelmsford, United Kingdom; University of Warmia, POLAND

## Abstract

The magnitude of zonular forces required to change the shape of the human lens while focusing at near; i.e., accommodating, is still under investigation. During accommodation, ciliary muscle contraction induces a large increase in lens central optical power (COP). Here we used finite element (FE) analysis to evaluate the correlation between zonular forces and lens surface curvatures, central thickness, COP, overall lens shape and longitudinal spherical aberration (LSA). Fresh isolated lenses from donors aged 20, 24, 26, and 30 years were the basis for the analyses. Lens nucleus elastic moduli were specified as equal to, 2, 3, 10, 20 and 30 times greater than its cortex. When equatorial zonular (Ez) force was increased in 3.125 x 10^-6^ N steps while the anterior zonular (Az) and posterior zonular (Pz) forces were decreased in 3.125 x 10^-6^ N steps, COP was evaluated. Independent of the increase in lens nuclear modulus, less than 0.02 N of Ez force was required to increase COP 10 diopters while Az and Pz forces were decreased. The lens peripheral surfaces flattened, central surfaces steepened, central lens thickness increased, COP increased and LSA shifted in the negative direction consistent with published *in vivo* accommodation studies. The minimal Ez force required to obtain 10 diopters of COP increase supports that increasing Ez force with decreasing Az and Pz force is the basis for the change in lens shape during accommodation. Since the COP increase was independent of increasing elastic modulus of the nucleus, stiffening of the lens nucleus is not the etiology of the universal age-related decline in accommodative amplitude that results in presbyopia in the fifth decade of life. Increased Ez zonular tension during accommodation has implications for the development and potential treatments of myopia, glaucoma, presbyopia, cortical cataracts and accommodative intraocular lens design.

## Introduction

Accommodation occurs as a result of a change in lens shape as demonstrated by Thomas Young in 1801 [1]. Central lens anterior and posterior surface curvatures and thickness increase while its peripheral surfaces flatten. These topographical changes cause an increase in central optical power (COP) and longitudinal spherical aberration (LSA) to shift in the negative direction [2]. The negative shift in LSA has been attributed to the peripheral lens surface flattening [3–5]. The purpose of the present study is to evaluate with finite element (FE) analysis the correlation between zonular force magnitude and these lens changes.

There have been many human lens FE analyses with most specifying the cortex elastic modulus greater than that of the nucleus [6–11]. This was based on measurements of lens elasticity using centrifugal forces (lens spinning) that provided the source material for computational modeling [9,12]. A lens nucleus with a lower elastic modulus than the cortex is not consistent with the physiochemical basis for differences in elastic moduli given that the nucleus has a higher refractive index, higher protein content and more disulfide bonds than the cortex [13,14]. In addition, Brillouin light scattering [15–18], optical coherent elastography [19,20], shear rheometry [21], probe penetration [22], bubble-based acoustic radiation force [23] and compression testing [24] demonstrate that the lens nucleus has the same or greater elastic modulus than the cortex.

As a consequence of FE models specifying the modulus of lens nucleus less than the cortex, a total ciliary muscle force >  0.060 N is required to obtain meaningful increases in lens COP [6–11]. This is exemplified by Burd’s et al. FE analysis [6] that predicted a zonular force between 0.080 N and 0.100 N that was significantly greater than the 0.015 N found by Fisher’s spinning lens experiment [12]. Ciliary muscle force has been measured [25]. Rhesus ciliary muscle strips of 5 mm coronal circular arc with meridional lengths of 4 mm, which is the total longitudinal muscle length, were placed in a force transducer. Carbachol, a supramaximal ciliary muscle stimulant [26], induced mean ± SEM coronal and longitudinal forces of 75 ± 28 mg and 94 ± 38 mg, respectively. By simply adding the coronal and longitudinal forces and 3 times the SEM, the maximum force one ciliary muscle strip can apply =  367 mg. Since the scleral diameter =  16 mm in the region of the ciliary muscle [27], approximately 10 ciliary strips would comprise the ciliary muscle circumference. Therefore,


Total Maximum Ciliary Muscle Force=10 × 367  mg=3.67 grams (1)

Consequently, a total maximum ciliary muscle of <  0.05 N (5 grams of force) is a conservative estimate.

When FE and mathematical analyses specified the nucleus elastic modulus equal to or greater than the cortex, ciliary muscle forces < 0.05N induced significant changes in lens COP [28–31]. In these FEM analyses, the most efficient increase in COP occurred when equatorial zonular force was increased while simultaneously anterior and posterior zonular forces were decreased. Peripheral lens surfaces flattened, central surfaces steepened, central thickness increased and COP increased.

**Fig 1:**
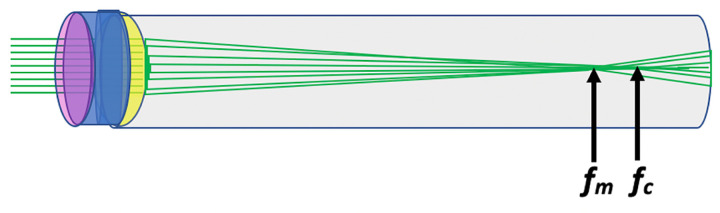
Schematic diagram of LSA. The focal point of marginal rays (*f*_*m*_) is closer to the back of a biconvex lens than the central/paraxial rays (*f*_*c*_). LSA is the difference between these focal points. When *f*_*m*_ is closer than *f*_*c*_ to the back of the lens, it is called positive spherical aberration and negative when it is further [34].

To avoid the lens nucleus modulus issue, zonular forces applied to the lens capsule without stroma were evaluated by FE analysis, force diagrams [32] and a balloon zonular force model [33]. These analyses demonstrated that to obtain the topographical changes observed during accommodation in human and rhesus monkey lens capsules void of lens stroma, the equatorial zonular (Ez) force must increase and anterior zonular (Az) and posterior zonular (Pz) forces simultaneously decrease. The peripheral surfaces flattened and the required total ciliary muscle force was < 0.02N. To assess whether these findings are consistent with zonular forces applied to the intact lens, the present FE analysis evaluated lens profile, COP and LSA (Fig 1) in response to increasing Ez force while Az and Pz forces were deceased. For this analysis the nuclear elastic modulus was equal to (L_0_), 2 times (L_2_) and 3 times (L_3_) greater than the cortex.

## Methods

### Geometric parameters and material properties

The sagittal profile x-y coordinates of fresh isolated human lenses from donors aged 20, 24, 26 and 30 years, kindly provided by Professors R.C. Augusteyn and A. Mohamed from their digital shadow photogrammetry study [35], formed the basis for the present analyses. A spline function was defined in a computer aided design software program (SolidWorks, version 2021) and then imported into a finite element mechanical program (Ansys Parametric Design Language, version 2021a) and fitted to the sagittal profile x-y coordinates of the lens from the 20-year-old donor [35]. For lenses from donors aged 24, 26, and 30 years [35], the sagittal profile x-y coordinates were fit with ellipses because they provided better fits than splines.

**Fig 2:**
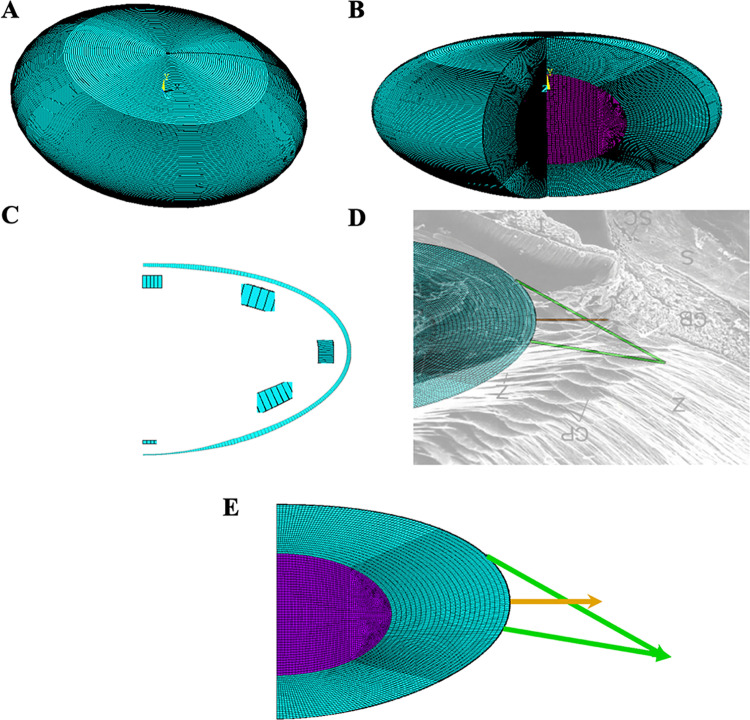
Finite element model. Anterior surface up of A) the whole lens, B) cut away to show the lens nucleus of the models with 2 and 3 times the modulus of the cortex since both of these models have the same number of elements, C) showing variation in capsular thickness with magnified views of each area, D) FE model lens superimposed on a scanning electron micrograph [36], to show the basis for the zonular angles, and E) direction of the Ez (orange arrow head) and the initial Az and Pz (green arrow head) forces on the lens.

Axisymmetric models were developed in Ansys using membrane elements (SHELL 209) for the lens capsule, axisymmetric plane elements (PLANE 183) for the lens stroma and quadrilateral membrane stiffness only elements (SHELL 208) for the zonules. The total number of finite elements for L_0_ was 6325 and for both L_2_ and L_3_ there were 5280 for the cortex and 3696 for the nucleus (Fig 2). The elastic modulus of the cortex was obtained from shear rheometry and optical coherent elastography studies [20,21]. The cortical elastic modulus for L_0,_ L_2_ and L_3_ =  150 Pa and for L_2_ and L_3,_ the nucleus elastic modulus was 300 Pa and 450 Pa, respectively.

Since the lens capsule varies in thickness [37,38], the Chien capsular thickness equation [38] was used with 220 elements (Fig 2c) and an elastic modulus of 0.3MPa [39,40]. The capsule and zonular fibers were considered as linear elastic, isotropic, and homogeneous material with Poisson’s ratios of 0.47 [41]. The zonules had an elastic modulus of 1.5 MPa [39,42].

The Ez had 1.6 mm length, 15 μm diameter, and were attached at the lens equator. Both the Az and Pz had diameters of 150 μm [36,43–45]. The Az had a length of 3.75 mm and was attached 1.0 mm (arc length) anterior to the equator as per the following formula for Az distance from the lens equator [46]:


$$Distance\;to\;E\;(mm)=0.0079\frac{mm}{years}\;age+0.202\;Ed(mm)-0.04\;AL(mm)$$ (2)

where *E* =  equator, Ed =  equatorial lens diameter =  8.34 mm, and AL =  axial length =  22.5 mm.

The Pz had a length of 3.00 mm and was attached 0.5 mm (arc length) from the lens equator. The Ez was in line with the lens equatorial plane and Az and Pz angled with respect to the horizontal to emulate the scanning electron microscopic image of the zonules as shown in Fig 2d. To simulate the origins of the Ez from the valleys of the anterior ciliary process and the Az and Pz from the pars plana, two separate forces were applied to the axisymmetric model on a 360-degree basis as shown by the orange and green arrows in Fig 2e.

### Boundary conditions

**Fig 3:**
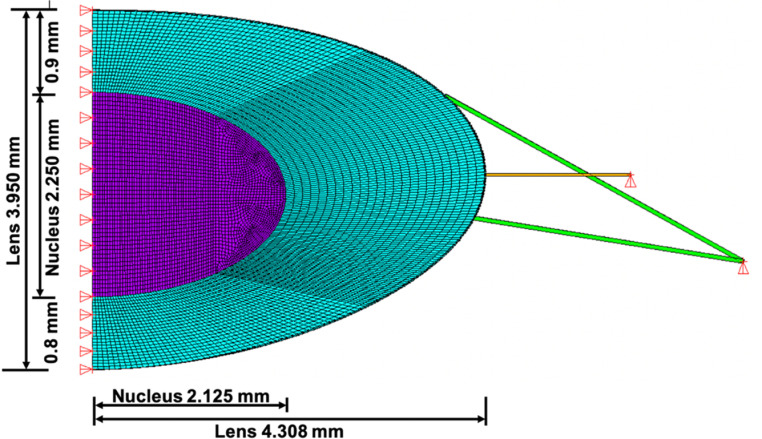
Dimensions of the lens and lens nucleus with FE analysis boundary conditions.

Considering that the lens is axisymmetric, the boundary conditions included restraining the central optic axis in the horizontal direction and rotational direction, the stromal elements were not permitted to rotate about the optic axis (Fig 3). The stretching endpoints of the zonules were constrained with translational degrees of freedom in the vertical direction and the rotational degrees of freedom about the optic axis (Fig 3). Nonlinear geometric static analyses were performed for all simulations.

## Application of zonular forces

When the eye is unaccommodated all the zonules are under tension, however, the applied ciliary muscle force is unknown. Since ciliary muscle dimensions of unaccommodated phakic and pseudophakic eyes are similar [47,48], a small total zonular force =  9.375 x 10^-5^ N defined the unaccommodated state. The equatorial zonular (Ez) force =  3.125 x 10^-5^ N and anterior (Az) plus posterior (Az) zonular force =  6.25 x10^-5^ N in all three models. Then Ez force was increased in 3.125 x 10^-6^ N steps while simultaneously the Az plus Pz force was decreased in 3.125 x 10^-6^ N steps until the maximum Ez force was equal to 0.03N. COP was calculated from the anterior (*r*_*a*_) and posterior (*r*_*p*_) radii of the best fitting sphere [49] circumscribing a 1 mm diameter of the lens vertices using the following thick lens formula [50]:


$$CentralOpticalPower\left(diopters\right)=\frac{n_{l}-n_{a}}{r_{a}}+\frac{n_{a}-n_{l}}{r_{p}}-\frac{t\left(n_{l}-n_{a}\right)\left(n_{a}-n_{l}\right)}{n_{l}r_{a}r_{p}}$$ (3)

where 𝑛_𝑎_ = 1.336 and nl = 1.42 are the indices of refraction of aqueous humor/vitreous and lens, and *t* =  central lens thickness.

## Longitudinal spherical aberration

**Fig 4:**
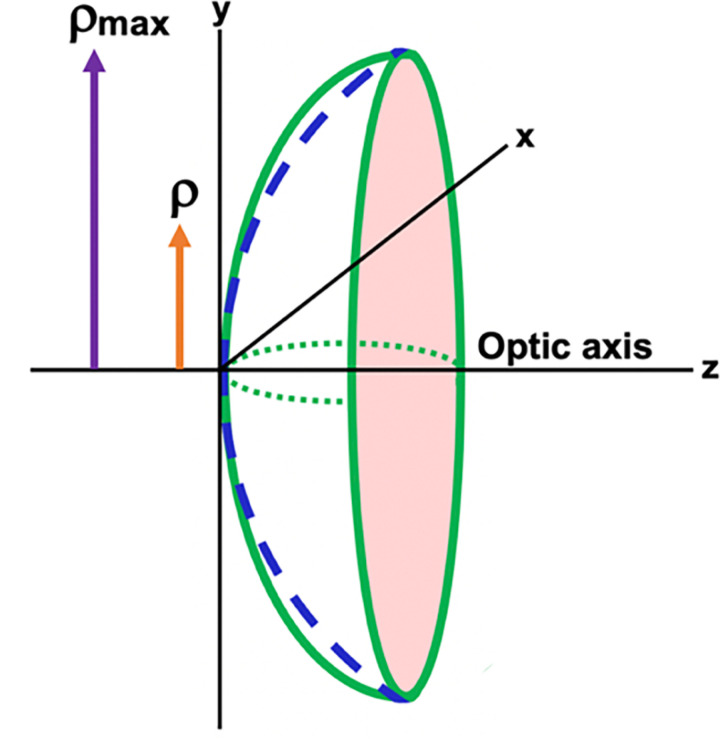
Coordinate system of the Forbes equation ( Eq 4) for fitting a curve to an aspheric surface (green line). ρ =  aperture radius and ρmax =  maximum aperture radius of the bfs (dashed blue line) [55].

From the FE model, lens profile coordinates were obtained as the COP was increased. The coordinates were imported into a software program (version 3.12.3, Python Software Foundation) and fit with the following Forbes aspheric equation [51–54]:


$$z(\rho )=\underset{\text{Part}1}{\underset{︸}{\frac{c_{bfs}\rho^{2}}{1+\sqrt{1-c_{bfs^{2}}\rho^{2}}}}}+\underset{\text{Part}2}{\underset{︸}{\frac{{\left(\frac{\rho }{\rho_{max}}\right)}^{2}\left(1-{\left(\frac{\rho }{\rho_{max}}\right)}^{2}\right)}{\sqrt{1-c_{bfs}^{2}\rho^{2}{\left(\frac{\rho }{\rho_{max}}\right)}^{2}}}\sum_{m=0}^{M}a_{m}Q_{m}^{bfs}{\left(\frac{\rho }{\rho_{max}}\right)}^{2}}}$$ (4)

where z =  sag (horizontal axis), *c*_*bfs*_ =  curvature of best fit sphere (bfs), *ρ* =  aperture radius of the bfs, *max *=  maximum aperture radius of the bfs, M =  maximum polynomial degree being used in the fit, $a_{m}Q_{m}^{bfs}{\left(\frac{\rho }{\rho_{max}}\right)}^{2}$ are polynomials of order 2 *m* *+ * 4 and configured so that the weighted root mean square slope of the departure along the normal is just the squares of $a_{m}$. The best fit sphere (bfs) for the Forbes equation captures the bulk of the lens shape (part 1 of Eq 4) with the Forbes aspheric terms (part 2 of Eq 4) describing the aspheric departure of the lens surface. The Forbes polynomials essentially describe the higher even order terms and the composite function describes the aspheric curve that fits the lens surface. The coordinate system for these parameters is shown in Fig 4.

Only the 6 mm diameter COZ lens profile coordinates were fit with Eq 4 because of the insertion of the anterior zonules and the steep curvature of the lens equator. The coefficients of Eq 4 were imported into an optical software program (OpTaliX-Pro v 12.0, Optical Engineering Software) for the lens surfaces of an optical eye model. The eye model had Navarro corneal and anterior chamber depth parameters [56]. For increasing lens optical powers of 2, 4, 5, 6, 8 and 10 diopters, a point source of light was moved respectively from 50 cm to 25 cm, 20 cm, 16.67 cm, 12.5 cm and 10 cm to the cornea. Spherical aberration for a 4 mm pupil was assessed to compare published results of the change in Zernike’s spherical aberration coefficient, $Z_{4}^{0}$, with increasing COP.

## Results

**Fig 5:**
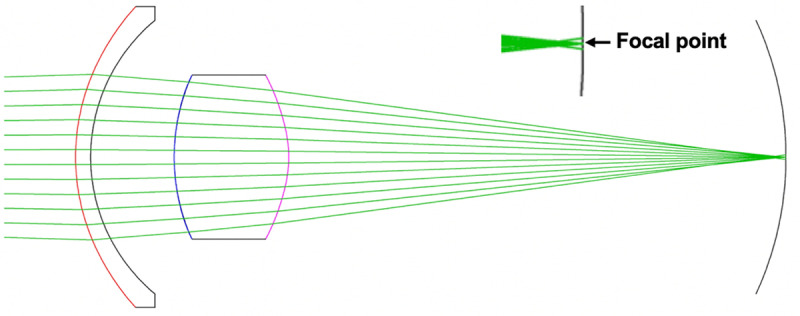
Optical model of the unaccommodated eye with Forbes aspheric curves ( Eq 4) representing the anterior and posterior lens surfaces. The magnified view of the focal point of parallel rays reveals positive spherical aberration.

The unaccommodated lens had a COP =  19.65 diopters, which was comparable to the 19.11 diopters of the Gullstrand’s schematic lens [57]. The focal point of the unaccommodated eye is shown in Fig 5.

## Effect of Zonular force

**Fig 6:**
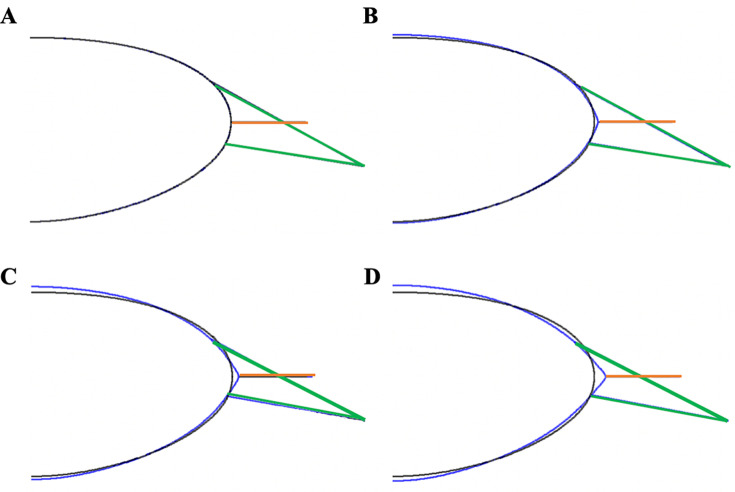
Lens profiles as Ez (orange lines) force was increased and Az and Pz (green lines) forces were decreased. A) unaccommodated state (black outline) and the COP increased (blue outline) by **B)** 3 diopters, **C)** 6 diopters and **D)** 10 diopters.

With increasing Ez force, equatorial diameter increased and peripheral surfaces flattened while the central surfaces steepened and central lens thickness and COP increased. The increase in central thickness and central surface steepening was greater anteriorly than posteriorly (Fig 6, and Supplemental Movie).

**Fig 7:**
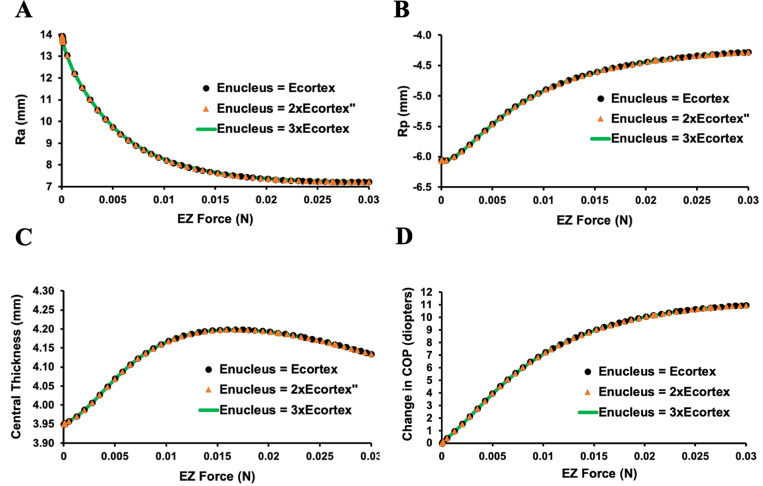
Graphs of the dimensional changes of the lens from the 20-year-old donor. A) Ra, B) Rp, C) central thickness and D) COP in response to increasing Ez force while Az and Pz forces were decreased.

**Fig 8:**
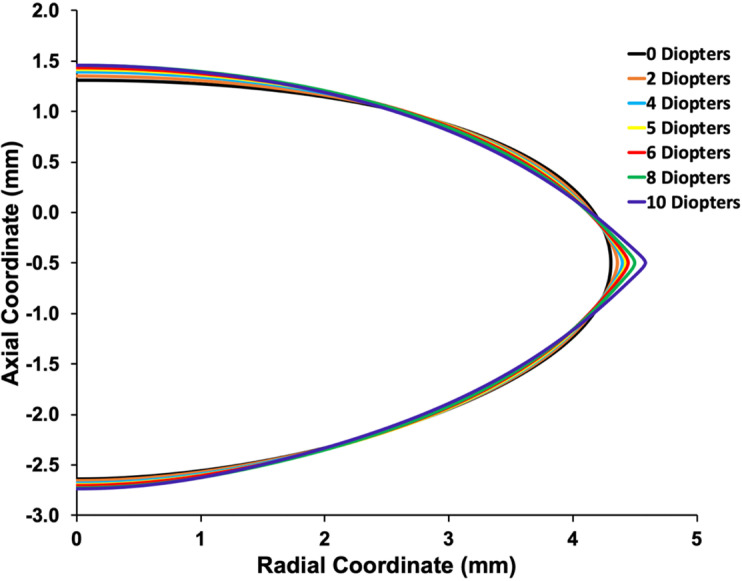
Graph of the change in shape of the lens from the 20-year-old donor. Equatorial zonular tension was increased while the peripheral surfaces flattened and central thickness increased more anteriorly than posteriorly without translation of the whole lens either anteriorly or posteriorly.

**Fig 9:**
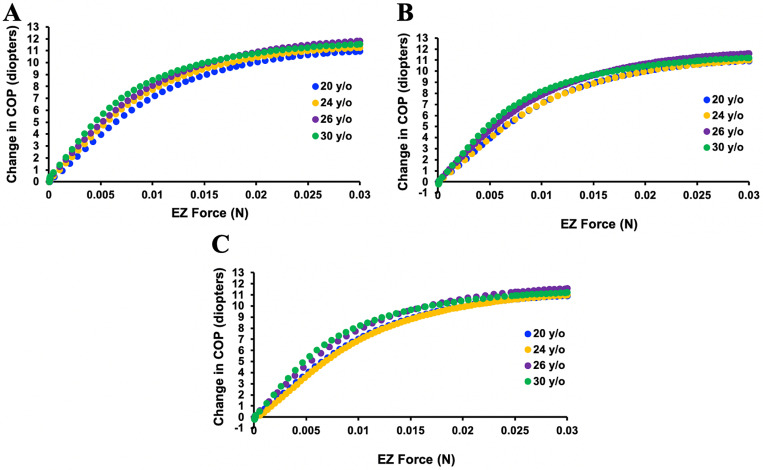
Graphs of the change in COP associated with increasing Ez force applied to each of the isolated lenses from donors of different age. When the lens nucleus elastic modulus was A) equal to that of its cortex, B) two times that of its cortex, and C) 3 times that of its cortex, there was no meaningful difference in their responses to the applied Ez force.

These lens parameters plateaued when Ez force was 0.017 N (Fig 7). For the same amount of Ez force, the change in Ra was more than the change in Rp (Fig 7a and b). Total central lens thickness increase plateaued at 0.017N and then began to slowly decrease (Fig 7c). The whole lens did not move anteriorly or posteriorly. Central lens thickness increased more anteriorly than posteriorly (Fig 8). These findings were consistent, regardless of the lens donor’s age or whether the lens nucleus was the same or 2 to 3 times greater than its cortex as exemplified by the association between COP and increasing Ez force shown in Fig. 9.

**Fig 10:**
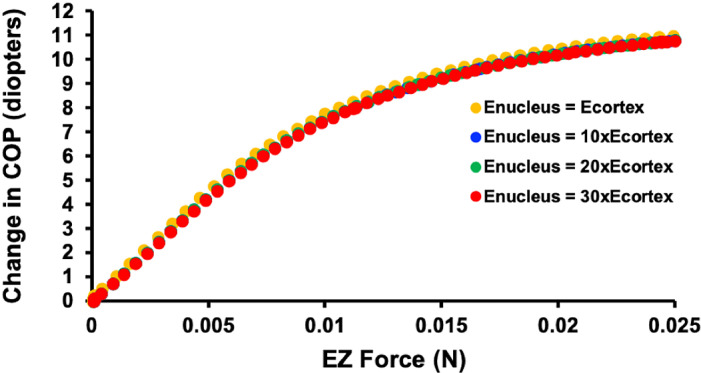
Graph showing the effect of increasing the elastic modulus of the nucleus 10, 20 and 30 times its cortex of the lens from the 24-year-old donor. The increase in COP associated with increasing Ez force was unaffected by these changes in the lens nucleus elastic modulus.

Since there was essentially no effect of increasing the elastic modulus of the lens nucleus on the change in COP, the effect of increasing it 10, 20 and 30 times on the lens from the 24-year-old donor was assessed. As shown in Fig 10, these large increases in the elastic modulus also did not affect the change in COP in response to increasing Ez force.

## Aspheric curve fits

**Fig 11:**
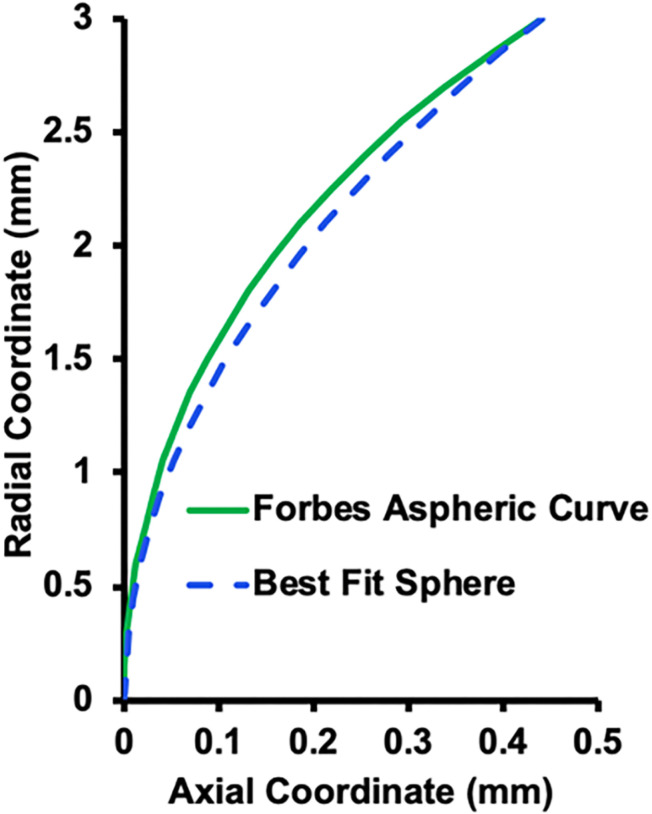
Forbes aspheric curve. Aspheric curve fit to the unaccommodated anterior lens surface (green line) with the bfs (dashed line blue) that was utilized for the fit.

Since there was no meaningful difference in the response to increasing Ez force between lenses from donors of different age, only the optical properties of the lens from the 20-year-old donor was assessed. The bfs was used to obtain the Forbes aspheric lens surface curve as shown in Fig 11. Note that only the Forbes aspheric lens surface curves were used to evaluate the optical properties of the model. The bfs was utilized to obtain the Forbes aspheric curves and was not used in the optical analyses. Mean ±  sd root mean square error (rmse) of the aspheric curve lens surface fits with change in COP from 0 to 10 diopters for Forbes polynomials of 10^th^, 15^th^ and 20^th^ degree are given in Table 1. Mean fits of the anterior and posterior lens surfaces for the 20^th^ degree Forbes polynomial were 6 times and 4 times better than the 10^th^ degree polynomial, respectively. Since radius of surface curvature is very sensitive to fit and can have significant optical effects, the 20^th^ degree Forbes equation polynomial was used in the optical analysis.

**Table 1:** Root mean square error of the Forbes Q^bfs^ curve fits (Eq 4) to the lenses.

Lens L_0_^a^ RMSE Fits (nm)						
Change COP	10^th^		15^th^		20^th^	
	Anterior	Posterior	Anterior	Posterior	Anterior	Posterior
0	4.0	14.3	1.4	5.8	0.6	3.1
2	2.3	1.9	0.7	0.5	0.4	0.3
4	1.9	3.9	0.5	0.9	0.3	0.4
5	1.8	5.6	0.5	1.5	0.3	0.8
6	1.7	7.3	0.4	2.2	0.2	1.3
8	1.6	10.2	0.4	3.5	0.2	2.3
10	1.1	13.1	0.3	5.2	0.1	3.7
Mean	2.0	8.0	0.6	2.8	0.3	1.7
STDEV	0.9	13.1	0.4	2.0	0.1	1.4
Lens L_2_^b^ RMSE Fits (nm)						
0	4.0	14.3	1.4	5.8	0.6	2.8
2	2.3	1.9	0.7	0.5	0.4	0.3
4	1.9	3.9	0.5	0.9	0.3	0.4
5	1.8	5.6	0.5	1.5	0.2	0.8
6	1.3	7.3	0.4	2.2	0.2	1.3
8	1.1	10.7	0.4	3.7	0.2	2.5
10	1.1	13.1	0.3	5.2	0.1	3.7
Mean	1.9	8.1	0.6	6.6	0.3	1.7
STDEV	1.0	4.7	0.4	3.4	0.1	1.3
Lens L_3_^c^ RMSE Fits (nm)						
0	3.9	14.3	1.4	5.7	0.6	2.8
2	1.8	1.9	0.7	0.5	0.4	0.3
4	1.4	4.1	0.5	1.0	0.3	0.4
5	1.8	5.6	0.5	1.5	0.3	0.8
6	1.3	7.4	0.4	2.3	0.2	1.4
8	1.6	10.5	0.4	3.7	0.2	2.5
10	1.1	13.2	0.3	5.2	0.1	3.8
Mean	1.8	8.2	0.6	2.8	0.3	1.7
STDEV	1.0	4.7	0.4	2.0	0.2	1.3

L_0_^a^ =  nucleus modulus is the same as that of the cortex; L_2_^b^ =  nucleus modulus is twice that of the cortex; L_3_^c^ =  nucleus modulus is three times that of the cortex.

**Fig 12:**
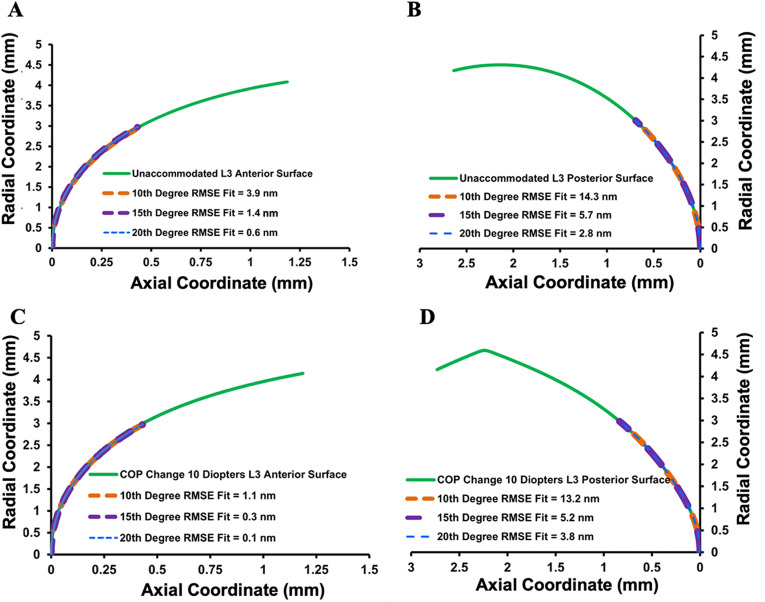
Forbes aspheric curve fits to L_3_ when unaccommodated. A) and B) and when the COP increased 10 diopters C) and D).

**Fig 13:**
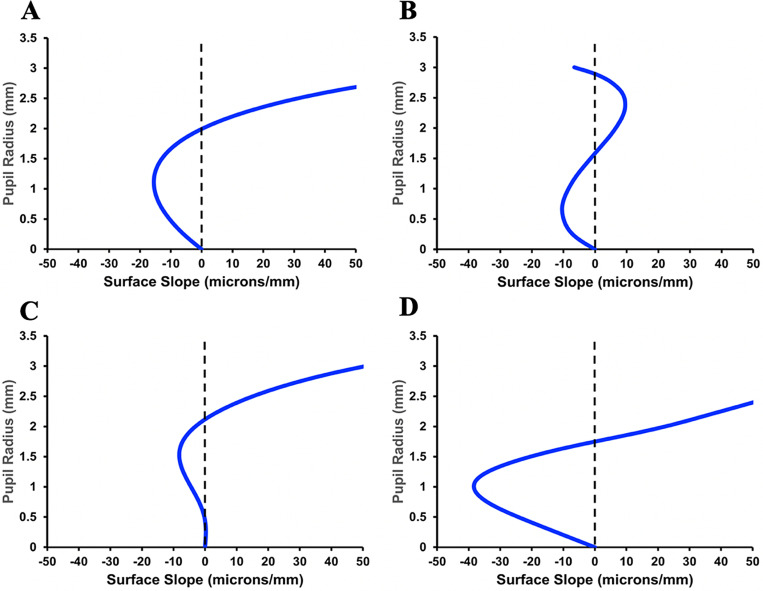
Local anterior and posterior aspheric deformation (residual after the bfs utilized for obtaining the Forbes aspheric curve was removed) surface slopes of the fitted curves to L_3_. Unaccommodated A) and B) and when the COP was increased 10 diopters C) and D), respectively.

Graphs of the aspheric curve fits to the lens surfaces are shown in Fig 12. In addition to the aspheric curves fitting the lens surfaces within nanometers, the curves were smooth as shown by the local surface slopes in Fig 13.

## Longitudinal spherical aberration

**Fig 14:**
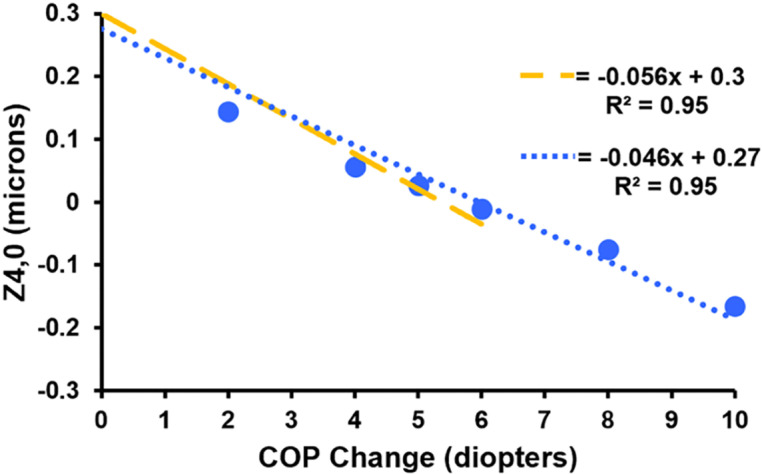
Graph demonstrating $Z_{4}^{0}$ **is indirectly linearly related to change in COP**. Yellow and blue dashed lines are regression analysis for changes in COP from 0 to 6 diopters and 0 to 10 diopters, respectively.

LSA shifted in the negative direction as demonstrated by $Z_{4}^{0}$ decreasing with slopes =  -0.056 (R^2^ =  0.95) and -0.046 (R^2^ =  0.95) microns/diopter for increases in COP from 0 to 6 and 0 to 10 diopters, respectively (Fig 14).

## Discussion

As observed during *in viv*o accommodation, the peripheral lens surfaces flattened, central surfaces steepened and central thickness and COP increased with increasing Ez force. There was no anterior or posterior movement of the whole lens. This is consistent with optical coherence tomography (OCT) studies that found no movement of the whole lens [58–60].

In the present FE analysis, the posterior zonule made a negative angle with respect to the equatorial plane based on the posterior zonule path that was identified from the electron microscopic scanning image of the zonules (Fig 2d). In prior FE analyses [6–11], the posterior zonules had a positive angle with respect to the equatorial plane. However, the results of the present study are essentially the same when compared to FEM analyses that had the lens nucleus elastic modulus the same or greater than that of the lens cortex [28,29]. This is expected since these analyses demonstrated Ez force most effectively increases COP [28].

Independent of whether the elastic modulus of the lens nucleus was the same, 2 times or 3 times that of the cortex, increasing Ez force less than 0.02N with decreasing Az and Pz forces, was sufficient to significantly increase COP to *in vivo* levels. Comparing the L_0_, L_2_ and L_3_ models, there was no significant difference in the change in RoC, central lens thickness, maximum COP or LSA in lenses from donors 20 to 30 years of age. This is consistent with the predictions of FE zonular force [32] and balloon [33] models of the lens capsule without lens stroma and FE and mathematical models of intact lenses that specified the nucleus elastic modulus to be the same or greater than that of the cortex [28–31].

The basis for comparing lenses with nuclear elastic moduli equal to, or 2 to 3 greater than the cortical modulus in the present study was to include the approximate range of elastic moduli of the study lenses from young donors [20,21]. This study was focused on zonular forces required for accommodation and not the etiology of the age-related decrease in accommodative amplitude that causes presbyopia in the fifth decade of life [61]. However, to assess the effect of increasing lens nuclear stiffness, the nucleus elastic modulus from the lens of the 24-year-old donor was increased 10, 20 and 30 times that of its cortex. The increase in COP associated with increasing Ez tension was unaffected by these changes. This is consistent with the lack of age-related change in peak longitudinal modulus of the nucleus [17].

Recently, the age-related decline in accommodative amplitude has been attributed to age-related increase in nucleus/cortex spatial ratio and not an increase in peak nucleus modulus [24]. However, swept-source scanning optical coherence tomography [62] and Scheimpflug imaging [63] demonstrate that the nucleus/cortex thickness and volume ratios actually decrease with age and the cortex increases at a rate approximately 3 times its nucleus [62]. Therefore, the basic premise of this hypothesis is not feasible. The incorrect nucleus/cortex spatial ratio was probably related to a consistent problem of omitting image registration from studies of the eye’s anterior segment. Image registration is a basic requirement of all measurements of the eye [64] including Brillouin light scattering [65,66]. This is exemplified by posterior segment optical coherence tomography as exquisitely manifested by optoretinography [64]. Consequently, alternate etiologies for the age-related decline in accommodative amplitude should be explored. For example, the continuous growth of the lens equatorial diameter [67] causing a decrease in ciliary muscle baseline length resulting in an age-related decline in the maximum force the ciliary muscle can apply [68,69].

Many previous FE analyses have contradicted the results of the present study [6–11]. However, all of these prior analyses assumed that an isolated lens without zonular tension had maximum COP. This was based on Helmholtz’s theory that during accommodation zonular tension decreases and COP increases [70]. Consequently, it was inferred that an isolated lens without zonular tension must have maximum COP emulating the *in vivo* fully accommodated state. However, when the ciliary muscle was disinserted in 24 cynomolgus monkeys, they became hyperopic, not myopic, and lost the ability to accommodate [71]. In addition, when RoCs of central optical zone diameters ≤  3 mm of isolated lenses were measured within 48 hours of the donor’s death, the RoCs were consistent with *in vivo* unaccommodated lenses [72]. Moreover parametric FEM analysis demonstrated that starting with isolated lenses with maximum COP; i.e., equivalent to the *in vivo* fully accommodated state, would require zonular forces and lens equatorial displacements that exceed physiological limits [28].

In the present analysis, the total zonular force to transition the isolated lens to the unaccommodated state was assumed to be 9.375 x 10^-5^ N. Consistent with this assumption, a recent *in vivo *study found that the largest mean zonular force for unaccommodated normal eyes with axial lengths of 22.0 to 24.5 mm was 8.66 x 10^-5^ ± 3.20 x 10^-5^ N [73].

We found that fresh isolated lenses had minimum COP as determined by curve fitting the shadow x-y profilometer coordinates, obtained from an independent study [35], and calculating the COP within the lens functional central optical zone diameter of  ≤  3 mm. Consequently, large COP increases occurred with Ez forces < 0.02N, which is well below the physiological limit of total ciliary muscle force [25] as noted in the introduction. Reading and near visual tasks require sustained ciliary muscle contraction and therefore the least amount of energy and force. Considering this basic requirement, the results of the present study are consistent with the mechanism of accommodation since large changes in COP occurred with a force significantly less than the maximum force the ciliary muscle can apply [25].

Peripheral surface flattening has been observed *in vitro* with zonular tension [74] and *in vivo* during accommodation [3–5]. Peripheral surface flattening can only occur if an outward force is applied to the lens equator. The indirect linear relationship between $Z_{4}^{0}$ and the change in COP demonstrates that peripheral lens surface flattening is the primary cause of the accommodative negative shift in spherical aberration. For a 4 mm pupil, the slope of $\frac{Z_{4}^{0}}{changeinCOP}$ =  -0.056 and -0.046 microns for a change in COP from 0 to 6 diopters and 0 to 10 diopters, respectively, which is comparable to *in vivo* measurements. Cheng et al. [75], Plainis et al. [76], Lopez-Gil et al. [77] and Atchison et al. [78] found slopes of -0.07, -0.054, -0.044 and -0.051 microns/diopter for accommodative changes of 0 to 6, 0 to 5, 0 to 4 and 0 to 3 diopters, respectively [79]. Consistent with these findings, theoretical aspheric surface models demonstrated surface asphericity affects LSA by an order of magnitude compared to the lens gradient [56,80].

The relatively smooth nanometer fits of the Forbes aspheric curves to the lens surfaces, which far exceed the approximate 30-micron fits of other equations [81,82] further supports the results. The anterior surface changed more than the posterior surface with increasing COP as observed *in vivo*. This is expected from a mechanical point of view because a small change in a flat surface induces a large radius of curvature change while a similar change in a steep surface would cause a much smaller radius of curvature change [68]. However, a difference in smoothness between the anterior and posterior was not anticipated. With increased COP; i.e., increased zonular force, the anterior lens surface became smoother as shown by the decrease in surface slope compared to the unaccommodated lens (Figs 13a and c). Interestingly, the opposite was true for the posterior surface. (Figs 13b and d). Consistent with these findings, the anterior surface rmse curve fits also improved with increasing COP, but this was not the case for the posterior surface (Table 1). There was a large improvement in posterior surface fit with the initial 2 diopter COP increase; however, with continued COP increase, the rmse fit decreased. Since the overall surface fits were excellent, these small surface changes would not affect lens optics, but they may give insight into lens biomechanics.

The differences in response of the anterior and posterior surface smoothness may be due to the difference in thickness of the anterior and posterior cortex, or a difference in adhesion of the cortical cells to the anterior and posterior capsule. The small differences in smoothness from increasing zonular force may be the basis for the higher incidence of posterior subcapsular cataracts compared to anterior capsular cataracts. On the other hand, less zonular force may cause a decrease in posterior cortical cell deformation which may lead to cataract formation. This may explain the observed association of increasing myopia with posterior subcapsular cataract and the higher incidence of cortical cataracts in high myopes [83]. Since myopes accommodate less because of their uncorrected closer focal point, there would be less zonular tension on the lens. The protein structure of lens cells has to be ordered to reduce light scattering for the lens to remain transparent [84]. Since the present analysis predicts there is constant zonular force on the lens throughout life that increases with accommodation, these forces would have to be part of the basic orientation of the endoplasmic reticulum (ER) and the Golgi apparatus and their function [85–87]. The ER and Golgi apparatus are located in the lens cortex, where cortical cataracts occur [88], A decrease in zonular tension may cause the ER and Golgi apparatus to significantly alter their configuration or function, which could lead to eventual misfolding of the lens structural proteins and consequent increased light scattering and cortical cataract formation [84]. Since the lens is derived from epithelial cells on its anterior surface that differentiate into lens fiber cells, the lens grows throughout life [67,89]. As the lens equatorial diameter increases, zonular force is decreased resulting in the increased incidence of cortical cataracts with age especially in the lens equatorial region [88].

In conclusion, only a small Ez force is required to induce a large increase in COP and induce the negative shift in spherical aberration observed during *in vivo* accommodation. Coupled with the stability of the lens [90,91], stress on the lens [92] and image registration studies that demonstrated the lens equator moves toward the sclera during accommodation [93,94], relaxation of all the zonules cannot be the basis for the mechanism of accommodation.

As first established by Thomas Young [1], only a change in lens shape is responsible for accommodation. During accommodation the cornea does not change shape [95,96]. Mean axial length increases 41  ±  17 microns [97]. Therefore, axial length maximally increases <  100 microns (mean plus 3 times sd), which does not meaningfully affect COP. The realization that the lens is solely responsible for accommodation is further supported by the comparable negative shift in $Z_{4}^{0}$ in the present study with *in vivo* measurements.

The increased Ez zonular tension during accommodation has implications for the development and potential treatments of myopia, glaucoma, presbyopia, cortical cataracts and accommodative intraocular lens design. Additionally, zonular force may play a role in posterior subcapsular formation.

## Supporting information

S1 File (Movie). (DOCX)

S1 Movie legend (MOV)
