# Supplementary material for: Finite element analysis of the lens profile during accommodation
Source: PLoS One. 2025 Mar 17;20(3):e0317740. doi: 10.1371/journal.pone.0317740 (PMC11913283; doi:10.1371/journal.pone.0317740)
Supplement: S1 File — (DOCX) [file pone.0317740.s001.docx]

**Supplement Legend**

**Supplement S1.** Movie of the changes in lens profile as equatorial zonular (Ez) force was increased and anterior (Az) and posterior (Az) zonular forces were simultaneously decreased with central optical power (COP) increased from the defined unaccommodated state to 10 diopters. Central anterior lens thickness increased more than the central posterior thickness while the peripheral surfaces flattened. There was no significant difference in profiles when the lens elastic nuclear modulus was the same, twice or three times that of the cortex. The Time code represents the seconds of computation.
